# Polypurine reverse-Hoogsteen (PPRH) oligonucleotides can form triplexes with their target sequences even under conditions where they fold into G-quadruplexes

**DOI:** 10.1038/srep39898

**Published:** 2017-01-09

**Authors:** Anna Solé, Emmanuelle Delagoutte, Carlos J. Ciudad, Véronique Noé, Patrizia Alberti

**Affiliations:** 1 Department of Biochemistry and Physiology, School of Pharmacy, University of Barcelona, Barcelona, Spain; 2 Structure et Instability of Genomes, Sorbonne Universités, Muséum National d’Histoire Naturelle, Inserm U 1154, CNRS UMR 7196, Paris, France

## Abstract

Polypurine reverse-Hoogsteen (PPRH) oligonucleotides are non-modified DNA molecules composed of two mirror-symmetrical polypurine stretches linked by a five-thymidine loop. They can fold into reverse-Hoogsteen hairpins and bind to their polypyrimidine target sequence by Watson-Crick bonds forming a three-stranded structure. They have been successfully used to knockdown gene expression and to repair single-point mutations in cells. In this work, we provide an *in vitro* characterization (UV and fluorescence spectroscopy, gel electrophoresis and nuclease assays) of the structure and stability of two repair-PPRH oligonucleotides and of the complexes they form with their single-stranded targets. We show that one PPRH oligonucleotide forms a hairpin, while the other folds, in potassium, into a guanine-quadruplex (G4). However, the hairpin-prone oligonucleotide does not form a triplex with its single-stranded target, while the G4-prone oligonucleotide converts from a G4 into a reverse-Hoogsteen hairpin forming a triplex with its target sequence. Our work proves, in particular, that folding of a PPRH oligonucleotide into a G4 does not necessarily impair sequence-specific DNA recognition by triplex formation. It also illustrates an original example of DNA structural conversion of a G4 into a reverse-Hoogsteen hairpin driven by triplex formation; this kind of conversion might occur at particular loci of genomic DNA.

Gene expression modulation and gene editing has been the focus of many researchers during the past years. Different synthetic oligonucleotide-based strategies have been developed over the years to target specific RNAs, genomic DNA sequences, proteins, and other cellular components^1^. These oligonucleotides include, for example, antisense oligonucleotides, small interfering RNA (siRNA), aptamers, triplex-forming oligonucleotides (TFO) able to bind to double-stranded DNA^2^,^3^,^4^, and peptide nucleic acids (PNA) able to recognise double-stranded DNA by strand displacement^5^,^6^. A number of genetic disorders are associated with a mutation in a single gene; different nucleic acid and nucleic acid analogues have been proven to be able to correct point mutations in genes. Among these oligonucleotides: single-stranded oligonucleotides^7^; chimeric RNA-DNA^8^; bifunctional triplex-forming oligonucleotides (TFBO) formed by a TFO domain and of a repair domain^9^,^10^; several PNA derivatives, such as pseudocomplementary PNAs (pc-PNA)^11^,^12^,^13^, bifunctional PNA-DNA conjugates (bis-PNA)^14^,^15^, tail-clamp PNAs (tc-PNA)^16^,^17^, and single-stranded oligodeoxynucleotides made of PNAs (PNA-ssODN)^18^,^19^. Beside oligonucleotides, targetable nucleases are also being studied to correct point mutations^20^,^21^.

Recently, our group succeeded in repairing single point mutations in cells using synthetic oligonucleotides that we named Polypurine reverse-Hoogsteen (PPRH) oligonucleotides^22^,^23^,^24^. PPRH oligonucleotides are non-modified DNA molecules composed of two mirror-symmetrical polypurine stretches separated by five thymines. They can fold, in principle, into hairpin structures where guanines pair with guanines and adenines pair with adenines via reverse-Hoogsteen base-pairing (•). They have been conceived to bind, via Watson-Crick base-pairing (x), to polypyrimidine sequences in genomic DNA by forming a three-stranded (or triplex) structure while maintaining their reverse-Hoogsteen hairpin conformation. These kinds of triplex structures, relying on G•GxC and A•AxT triplets, are often referred to as “purine-motif triplexes”. Their formation requires the presence of divalent cations, such as Mg^2+^ and is relatively independent of pH^25^,^26^,^27^. Purine-motif triplexes may be observed at high temperatures, melting only upon disruption of their Watson-Crick duplex template^28^. In previous works, we described the ability of PPRH oligonucleotides to bind to either the template or the coding strand of the double-stranded DNA target^29^,^30^, causing strand displacement. PPRH oligonucleotides knock down the expression of target genes with several advantages, such as stability, low immunogenicity and cost, compared to other approaches^31^,^32^. PPRH oligonucleotides have been used for gene silencing^29^,^30^,^33^,^34^ and, recently, for gene targeting to repair single-point mutations^23^,^24^. Repair-PPRH oligonucleotides are formed by a PPRH motif bearing an extended DNA sequence homologous to the sequence to be repaired but containing the correct nucleotide (Fig. 1).

Our interest now is to carefully investigate the secondary structures and stabilities of PPRH and repair-PPRH oligonucleotides and of the complexes they form with their single-stranded target sequences. We are particularly interested in elucidating the behaviour of PPRH oligonucleotides bearing runs of consecutive guanines. When designing a PPRH motif, it is important to take into consideration its possible high guanine content. The presence of runs of consecutive guanines can impair folding into a reverse-Hoogsteen hairpin conformation in favour of a guanine-quadruplex (G4) structure, especially under physiological conditions. G4s are four-stranded nucleic acid structures formed by the stacking of tetrads of hydrogen-bonded guanines^35^. Differently from duplex or triplex structures, the stability of G4s depends on the nature of the cation present in solution; in particular they are strongly stabilized by the physiological relevant potassium ion.

In a previous work, we designed and used two repair-PPRH oligonucleotides, named *HpE6rep6* and *HpE2rep2*, to correct single-point mutations in the dihydrofolate reductase (*dhfr*) gene in mammalian cell lines (in exons 6 and 2, respectively)^23^. Each of the two repair-PPRH oligonucleotides, *HpE6rep6* and *HpE2rep2*, is composed of a PPRH motif (*HpE6* and *HpE2*, respectively) designed to target a polypyrimidine/polypurine tract next to the *dhfr* gene sequence to be repaired, and of a 25 nucleotide single-stranded extension (*rep6* and *rep2*, respectively) homologous to the sequence to be repaired (Table 1). *HpE6* and *HpE2* motifs have very different guanine contents. *HpE6* bears only two runs of two consecutive guanines and can in principle fold into a reverse-Hoogsteen hairpin (as illustrated in Table 1). *HpE2* contains eight runs of two consecutive guanines and can in principle fold either into a reverse-Hoogsteen hairpin (as illustrated in Table 1) or into a G4 structure. In this work we provide an *in vitro* characterization of the structures and stabilities of *HpE6* and *HpE2* motifs (alone and with their *rep* extensions) and of the complexes they form with their single-stranded target sequences. Our work reveals unexpected behaviours of these two PPRH motifs. In particular we show that *HpE2* folds, in potassium, into a stable G4; nevertheless, in the presence of its single-stranded polypyrimidine target sequence, it converts into a reverse-Hoogsteen hairpin and forms a triplex. Besides elucidating the structure of the two repair-PPRH oligonucleotides and of the complexes they form with their targets, our work proves that folding of a PPRH oligonucleotide into a stable G4 does not necessarily impair sequence-specific DNA recognition by triplex formation. “DNA comes in many forms”^36^, our work also illustrates an original example of DNA structural conversion of a G4 into a reverse-Hoogsteen hairpin driven by triplex formation. We suggest that this kind of conversion might spontaneously occur at particular loci of genomic DNA and be involved in genome dynamics.

## Results and Discussion

### Structure and stability of *HpE6* and *HpE2* motifs

Given their sequences, the *HpE6* motif can potentially fold into a 23 base-pair reverse-Hoogsteen hairpin structure with three pyrimidine base-pair interruptions (as illustrated in Table 1), while the *HpE2* motif can potentially fold into a 13 base-pair reverse-Hogsteen hairpin structure (as illustrated in Table 1) or into a G4 structure. To gain insight into the structures formed by *HpE6* and *HpE2* oligonucleotides and their stabilities, we carried out an investigation by UV-absorption and fluorescence spectroscopy under different ionic conditions (100 mM NaCl or KCl, in the absence or in the presence of 10 mM MgCl_2_) and by non-denaturing polyacrylamide gel electrophoresis (PAGE).

As expected for a double-stranded structure, *HpE6* melting profiles were independent of the nature of the monovalent cation, Na^+^ or K^+^, present in solution, and strongly depended on the presence of Mg^2+^ (Supplementary Fig. S1): in the absence of magnesium, *HpE6* did not form a stable structure, its temperature of thermal transition *T*_t_, (defined in the Methods section) was lower than 20 °C; whereas in the presence of magnesium, *HpE6* formed a structure with a *T*_t_ of 33 °C. The independence of *T*_t_ of strand concentration (2 and 20 μM) supported folding of *HpE6* into an intramolecular structure (Supplementary Fig. S1). Overall, these data support folding of *HpE6* into a reverse-Hoogsteen hairpin structure in the presence of Mg^2+^. The addition of the *rep* extension did not affect the stability of the *HpE6* hairpin: the normalized melting profile of *HpE6rep6* was identical to the normalized melting profile of *HpE6* (Supplementary Fig. S2).

**Figure 2:**
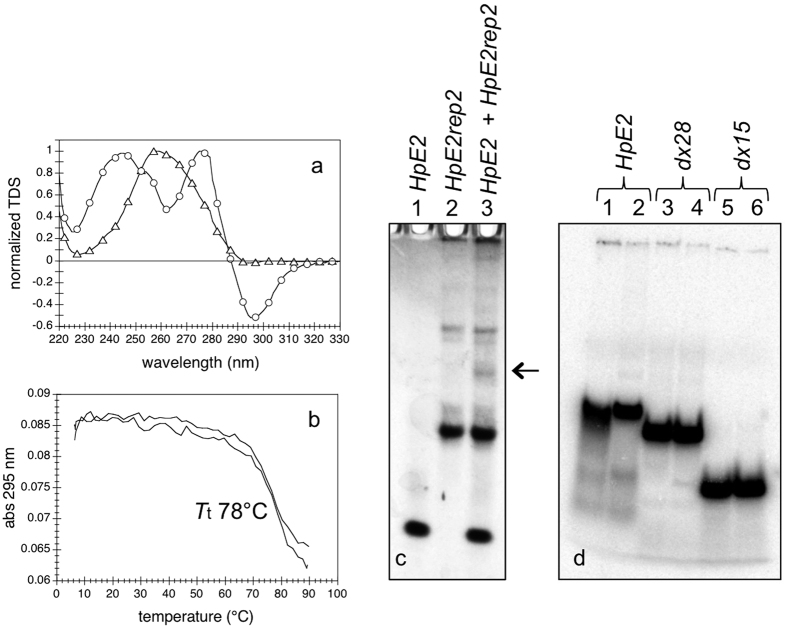
Structures of *HpE2* in sodium and in potassium. (**a**) Normalized TDS of *HpE2* in 100 mM NaCl + 10 mM MgCl_2_ (triangles) and in 100 mM KCl + 10 mM MgCl_2_ (circles). (**b**) Absorbance at 295 nm as a function of temperature of 2 μM *HpE2* in 100 mM KCl + 10 mM MgCl_2_. We verified by circular dichroism that *HpE2* was completely unfolded above 90 °C (Supplementary Fig. S3). (**c**) Non-denaturing PAGE of 50 μM *HpE2* (lane 1), 50 μM *HpE2rep2* (lane 2) and a mix of 50 μM *HpE2* + 50 μM *HpE2rep2* (lane3) in potassium. Samples were prepared in a buffer containing 100 mM KCl, heated at 90 °C for 2 min and slowly cooled at 4 °C. The gel and the migration buffer contained 20 mM KCl. Oligonucleotides were detected by UV-shadow. (**d**) Non-denaturing PAGE of *HpE2* at 2 and 20 μM strand concentration (lanes 1 and 2, respectively); *dx28* and *dx15* are two intermolecular Watson-Crick duplexes of 28 and 15 base-pair length, respectively (1 μM of radiolabelled strand and 1.5 μM of non-radiolabelled complementary strand in lanes 3 and 5; 10 μM of radiolabelled strand and 15 μM of non-radiolabelled complementary strand in lanes 4 and 6; radiolabeled strands were at nanomolar concentrations). Samples were prepared in a buffer containing 100 mM NaCl + 10 mM MgCl_2_, heated at 90 °C for 2 min and slowly cooled at 4 °C. Both the gel and the migration buffer contained 20 mM NaCl and 10 mM MgCl_2_.

Contrary to *HpE6*, the structure of *HpE2* depended on the nature of the monovalent cation, as revealed by Thermal Difference Spectra (TDS) (Fig. 2a). A TDS is obtained by subtracting the absorbance spectrum at low temperature (where the oligonucleotide is in its folded state) from the absorbance spectrum at high temperature (where the oligonucleotide is in its unfolded state). TDS have a specific shape for each type of nucleic acid structure, and TDS are very similar within a nucleic acid structural family^37^. In potassium, *HpE2* exhibited a TDS typical of a G4 structure, with two positive maxima around 245 and 275 nm and a negative minimum around 295 nm, while in sodium it did not form a G4 (Fig. 2a).

The G4 structure formed by *HpE2* in potassium displayed a temperature of thermal transition *T*_t_ of 78 °C (Fig. 2b). The addition of the *rep* extension did not affect the stability of the *HpE2* G4: the normalized melting profile of *HpE2rep2* was identical to the normalized melting profiles of *HpE2* (Supplementary Fig. S2). To investigate the molecularity of the G4 formed by the *HpE2* domain in potassium, we carried-out a non-denaturing PAGE experiment (Fig. 2c). *HpE2* migrated as a single band (Fig. 2c, lane 1); the main band of *HpE2rep2* migrated slowly compared to *HpE2* (Fig. 2c, lane 2). To determine whether *HpE2* band and *HpE2rep2* main band corresponded to intra- or intermolecular G4s, we annealed a mix of *HpE2* and *HpE2rep2* at equimolar strand concentrations (Fig. 2c, lane 3): the migration pattern displayed two major bands with the same mobility of *HpE2* and *HpE2rep2* alone, and a third minor band of slower mobility (indicated in Fig. 2c with an arrow). The absence, in the migration pattern of the annealed mix *HpE2* + *HpE2rep2*, of bands with mobility intermediate to the mobility of *HpE2* and *HpE2rep2* alone and the appearance of a minor band of slower mobility indicate that the *HpE2* and *HpE2rep2* major bands are intramolecular structures. The intermolecular species formed by the association of *HpE2* with *HpE2rep2* (indicated with an arrow in lane 3 of Fig. 2c) constituted a very minor fraction, despite the relatively high strand concentration used in this PAGE experiment (50 μM of each strand).

In sodium, *HpE2* folded into a non-G4 structure whose stability increased in the presence of magnesium (*T*_t_ of 33 °C in the absence of Mg^2+^, *T*_t_ of 39 °C in the presence of Mg^2+^, at 2 μM strand concentration) (Supplementary Fig. S4). Differently from *HpE6*, the *T*_t_ of *HpE2* in sodium increased with increasing strand concentration (Supplementary Fig. S4), demonstrating the formation of intermolecular species. Non-denaturing PAGE of *HpE2* and of two control duplexes of 28 and 15 base-pairs in sodium supported that, at 2–20 μM strand concentration, the major structure of *HpE2* was not the 13 base-pair hairpin but the 31 base-pair intermolecular duplex with 5 thymine base-pair interruptions (Fig. 2d).

In conclusion, *HpE6* and *HpE2* behave differently. The *HpE6* motif (alone as well as with its *rep6* extension) folds into a reverse-Hoogsteen hairpin. The structure of the *HpE2* motif is cation-dependent: in potassium, *HpE2* (alone as well as with its *rep2* extension) mainly folds into a stable intramolecular G4 (by stable, we mean with a temperature of thermal transition far above the physiological temperature of 37 °C), while in sodium *HpE2* folds into a reverse-Hoogsteen intermolecular duplex.

We next investigated whether *HpE6* and *HpE2* motifs, alone and with their *rep* extensions, formed triplexes with their single-stranded polypyrimidine target sequences under salt conditions mimicking the intracellular environment, where *HpE6rep6* and *HpE2rep2* oligonucleotides were addressed in previous gene correction experiments^23^,^24^, *i*.*e*. in the presence of potassium and magnesium (magnesium is also generally required to form three-stranded structures of purine motif).

### Structure of the *HpE6rep6* + *Y6rep6*’ complex

To verify if the reverse-Hoosgteen hairpin *HpE6* motif formed a triplex structure with its single-stranded polypyrimydine target, we first carried out UV-melting and PAGE experiments (Supplementary Fig. S5). Both of these approaches indicated that the complex formed by *HpE6rep6* and *Y6rep6*’ had the same stability of the duplex formed by *Y6rep6*’ and its complementary strand *R6rep6* (oligonucleotide sequences are reported in Table 1). Nevertheless they did not allow inferring triplex formation, since they did not allow discriminating between a triplex structure and a duplex structure with a hanging third strand.

**Figure 3:**
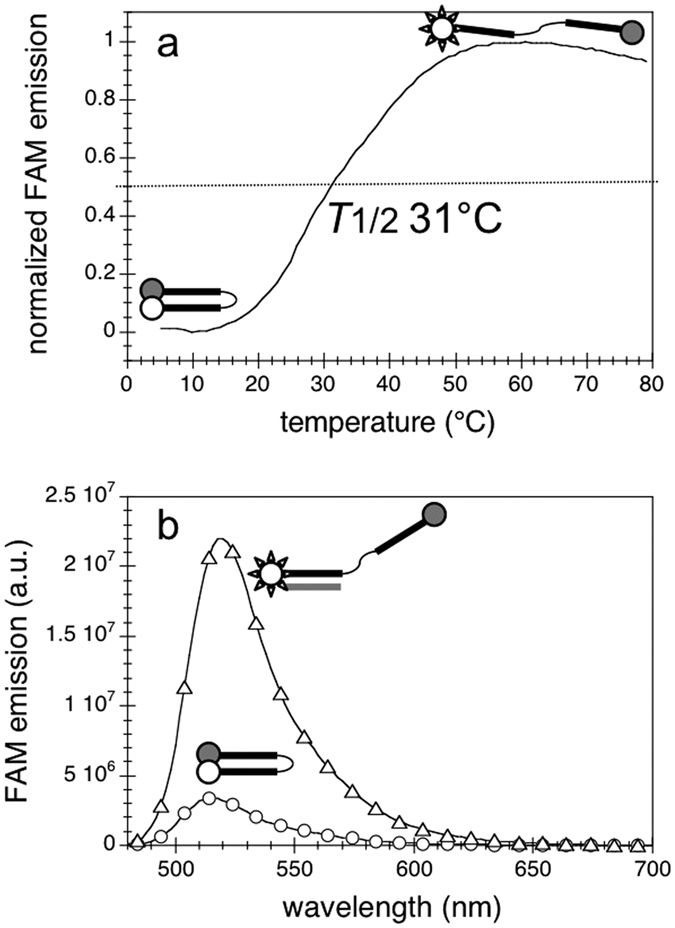
Structure of FAM-*HpE6*-Dabcyl alone and in the presence of its target *Y6*. (**a**) Normalized FAM emission of 0.2 μM FAM-*HpE6*-Dabcyl; excitation wavelength 470 nm, emission wavelength 520 nm. (**b**) Emission spectra at 5 °C of 0.2 μM FAM-*HpE6*-Dabcyl alone (circles) and upon addition of 1 μM *Y6*; excitation wavelength 470 nm. The intensity of FAM-*HpE6*-Dabcyl emission in the presence of *Y6* was similar to the intensity of FAM-*HpE6*-Dabcyl at 80 °C, where the oligonucleotide was completely unfolded. Measurements were run in a buffer containing 100 mM KCl and 10 mM MgCl_2_. White and grey symbols in the schematic illustration of the formed structures represent FAM dye and Dabcyl quencher, respectively.

To ascertain triplex formation, we designed a fluorescent *HpE6* bearing a 6-carboxyfluorescein (FAM) dye at its 5′ end and a Dabcyl quencher at its 3′ end, named FAM-*HpE6*-Dabcyl. When the oligonucleotide is folded into a hairpin, the FAM fluorescence is quenched by the Dabcyl quencher; when the oligonucleotide is in an open state the FAM fluorescence is restored. We first recorded the fluorescence of FAM-*HpE6*-Dabcyl alone as a function of temperature (Fig. 3a). The melting profile followed by FAM fluorescence showed that the double-labelled oligonucleotide folded into a structure with a temperature of half-dissociation *T*_1/2_ (defined in the Methods section) of 31 °C (Fig. 3a), in accordance with the temperature of thermal transition determined by UV-melting for the non-labelled *HpE6 (T*_t_ of 33 °C) (Supplementary Fig. S1). Nevertheless, when the polypyrimidine target Y6 was added to the FAM-*HpE6*-Dabcyl solution at 5 °C (*i*.*e*. at a temperature where the double-labelled oligonucleotide was completely folded into a hairpin structure), the FAM fluorescence was completely restored (Fig. 3b). This reveals that *HpE6* hybridizes to its pyrimidine target *Y6* but does not form a triplex. Hence, despite its reverse-Hoogsteen hairpin structure, *HpE6* did not form a triplex with its polypyrimidine target, but a duplex with a hanging third strand.

In conclusion, the hairpin-prone *HpE6* motif recognizes its polypyrimidine target (*i*.*e. HpE6* binds to *Y6*), nevertheless the formed complex is not a triplex: one portion of the *HpE6* motif form a Watson-Crick duplex with *Y6*, and the other portion hangs in an open state. The three-pyrimidine base-pair interruptions in the reverse-Hoogsteen hairpin structure might be detrimental to triplex formation^32^.

### Structure of the *HpE2rep2* + *Y2rep2*’ complex in potassium

In potassium the *HpE2* motif (alone or with the *rep2* extension) formed a stable G4. Electrophoretic mobility shift assay (EMSA) revealed that, despite its G4 structure, the *HpE2* oligonucleotide bound to its polypyrimidine target *Y2* (Supplementary Fig. S6). In order to gain insight into the complex(es) formed by the *HpE2* motif in the *rep* context, we compared UV-melting profiles of *HpE2rep2* + *Y2rep2*’ with melting-profiles of two different DNA constructs: (i) the duplex formed by *Y2rep2*’ with its complementary strand *R2rep2*, and (ii) the double-stranded/single-stranded structure formed by *Y2rep2*’ with the shorter oligonucleotide *rep2* (Table 1).

**Figure 4:**
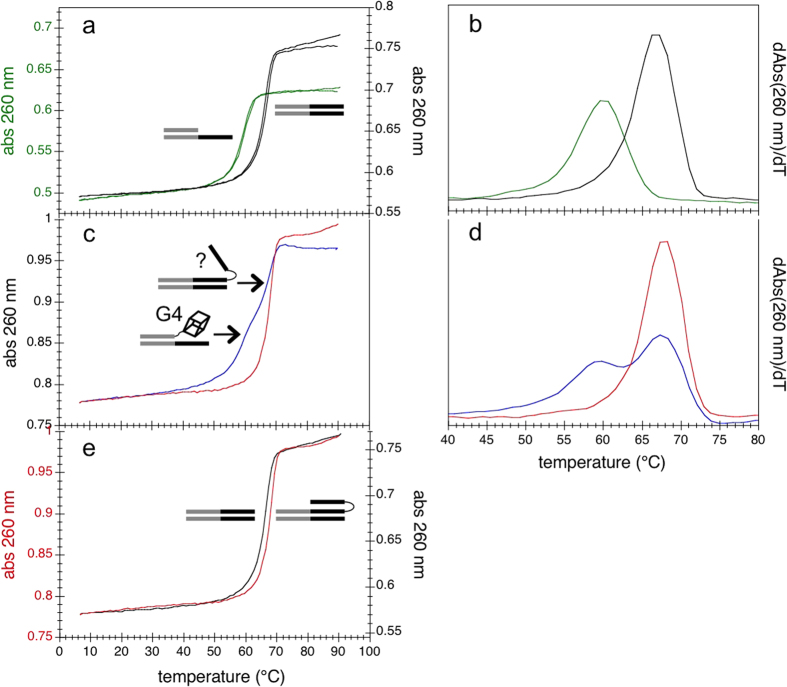
UV-melting profiles of the complex(es) formed by *HpE2rep2* and *Y2rep2*’ in potassium and magnesium. Absorbance at 260 nm as a function of temperature of different DNA constructs. (**a**) *Y2rep2*’ + *R2rep2* (black) and *Y2rep2*’ + *rep2* (green) (both cooling and heating profiles are shown); (**b**) first derivative of the absorbance at 260 nm as a function of temperature of cooling profiles shown in (**a**). (**c**) *Y2rep2*’ + *HpE2rep2*, when cooling from 95 to 5 °C (blue) and when heating from 5 °C to 95 °C (red); (**d**) first derivative of the absorbance at 260 nm as a function of temperature of profiles shown in (**c**). (**e**) *Y2rep2*’ + *R2rep2* (black) and *Y2rep2*’ + *HpE2rep2* (red) when heating from 5 °C to 95 °C. Each oligonucleotide was at 1 μM strand concentration. Measurements were run in a buffer containing 100 mM KCl and 10 mM MgCl_2_, at a temperature scan rate of 0.2 °C/min.

**Figure 5:**
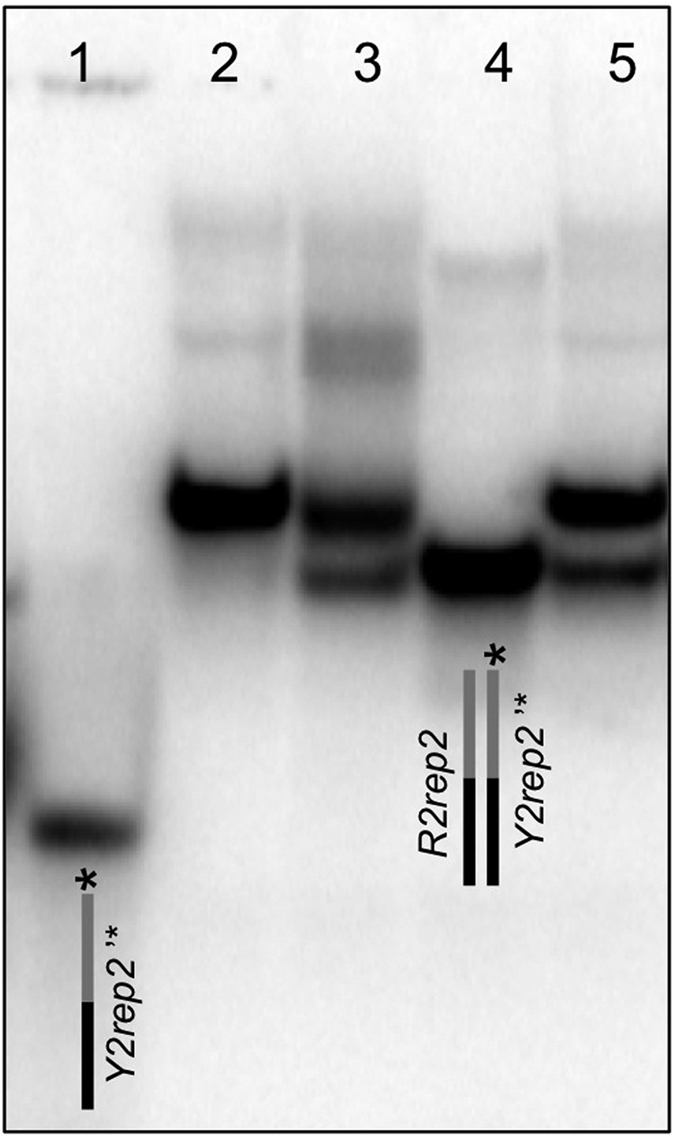
PAGE of the complex(es) formed by *HpE2r*ep2 + Y2r*ep2*’ in potassium and magnesium. Electrophoretic mobility shift assay of radiolabeled *Y2rep2*’ oligonucleotide (*Y2rep2*’*). Lane 1: *Y2rep2*’*. Lane 2: *HpE2rep2* + *Y2rep2*’*, the mix was heated at 95 °C and slowly cooled at 5 °C. Lane 3: *HpE2rep2* was heated at 95 °C and slowly cooled at 5 °C, then *Y2rep2*’* was added. Lane 4: *Y2rep2*’* plus its complementary strand R2rep2. Lane 5: *Y2rep2*’* plus equal amounts of *HpE2rep2* and *R2rep2*; the mix was heated at 95 °C and slowly cooled at 5 °C. Samples were annealed in 100 mM KCl + 10 mM MgCl_2_. Both the gel and the migration buffer contained 20 mM KCl and 10 mM MgCl_2_; migration was carried out at about 15 °C.

The 38 base-pair duplex formed by *Y2rep2*’ + *R2rep2* and the 25 base-pair duplex portion formed by *Y2rep2*’ + *rep2* exhibited a single thermal transition at 66 °C and 60 °C, respectively (Fig. 4a and b). When annealing *HpE2rep2* + *Y2rep2*’ from high to low temperature, two transitions were observed: one at 67–68 °C and one at 59–60 °C (Fig. 4c and d, blue curves), supporting the formation of two distinct complexes. The *T*_t_ of the first transition was 1–2 °C higher than the *T*_t_ of the long duplex formed by *Y2rep2*’ + *R2rep2* (about 66 °C). This transition is consistent with the formation of an extended complex where *HpE2rep2* hybridizes to *Y2rep2*’ all along its length (as illustrated in Fig. 4c). The *T*_t_ of the second transition was identical to the *T*_t_ of the short duplex formed by *Y2rep2*’ + *rep2* (about 60 °C). This transition is consistent with the formation of a complex where only the *rep2* domain of *HpE2rep2* hybridizes to *Y2rep2*’, while the *HpE2* motif forms a hanging G4 structure (as illustrated in Fig. 4c). A rough graphical analysis of the UV-melting profile of the *HpE2* motif in potassium plus magnesium (Fig. 2b) allows understanding this behaviour. Above about 65 °C, the *HpE2* motif is in equilibrium between a G4 form and an unstructured form (Fig. 2b), hence, at temperature above ≈65 °C, the unfolded fraction of *HpE2rep2* can hybridize all along the *Y2rep2*’ strand (and the free strand of the *HpE2* motif can potentially form a triplex structure). Below about 65 °C, the *HpE2* motif is completely folded into a G4 (Fig. 2b), hence, at temperature below ≈65 °C, *HpE2rep2* can hybridize to *Y2rep2*’ only along its *rep2* domain. Subsequent heating of *HpE2rep2* + *Y2rep2*’ from low to high temperature resulted in a single transition at 68 °C (Fig. 4c and d, red curves). This supports that, after annealing *HpE2rep2* + *Y2rep2*’ from high to low temperature, a single extended complex is present where *Hp2rep2* is hybridized to *Y2rep2*’ all along its length. Overall, UV-melting profiles support the following folding pathway for the complex *HpE2rep2* + *Y2rep2*’. When annealing from high to low temperature, two distinct complexes are formed: an extended complex and a less stable duplex/G4 complex (as illustrated in Fig. 4c); however, during the annealing process, the duplex/G4 complex converts to the extended complex. In agreement with a structural conversion, the detection of duplex/G4 complexes depended on the temperature scan rate: upon annealing from high to low temperature at a slower temperature scan rate, the 60 °C transition could not be resolved anymore (Supplementary Fig. S7). This means that the conversion from the duplex/G4 structure to the extended structure is sufficiently slow to be observed at a temperature scan rate of 0.2 °C/min but sufficiently fast to be no more observed at slower temperature scanning rates. The presence of a single complex after annealing from high to low temperature, was confirmed by EMSA. Indeed, when *HpE2rep2* and radiolabelled *Y2rep2*’ (*Y2rep2*’*) were slowly annealed together from high to low temperature, a single band was observed (Fig. 5, lane 2). Differently, when *Y2rep2*’* was added, at low temperature, to a pre-structured *HpE2rep2 (i. e*. cooled alone from high to low temperature in the presence of potassium), two distinct complexes were formed (Fig. 5, lane 3): a major complex migrating as the complex formed by *HpE2rep2* + *Y2rep2*’* upon annealing from high to low temperature (Fig. 5, lane 2) (which is an extended complex, as supported by UV-melting), and a minor faster-migrating complex that may correspond to a duplex/G4 structure.

Both UV-melting and EMSA suggested that the extended complex formed by *HpE2rep2* + *Y2rep2*’ was more stable than the duplex formed by *R2rep2* + *Y2rep2*’. Indeed, in UV-melting experiments, the heating profile of *HpE2rep2* + *Y2rep2*’ was shifted of 1–2 °C toward higher temperatures compared to the heating profile of the long duplex *Y2rep2*’ + *R2rep2* (Fig. 4e) (UV melting profiles were perfectly reproducible). Consistently, in EMSA, when *Yrep2*’* was annealed from high to low temperature in the presence of both *HpE2rep2* and *R2rep2* in equal amounts, two bands of different intensities appeared (Fig. 5, lane 5): the major band had the same mobility of the extended complex formed by *HpE2rep2* + *Y2rep2*’ (Fig. 5, lane 2), while the minor band migrated as the duplex formed by *Y2rep2*’ and *R2rep2* (Fig. 5, lane 4). Overall these results suggest that in the extended complex the third strand of the *HpE2* motif is not hanging, but forms a stabilizing triplex structure.

**Figure 6:**
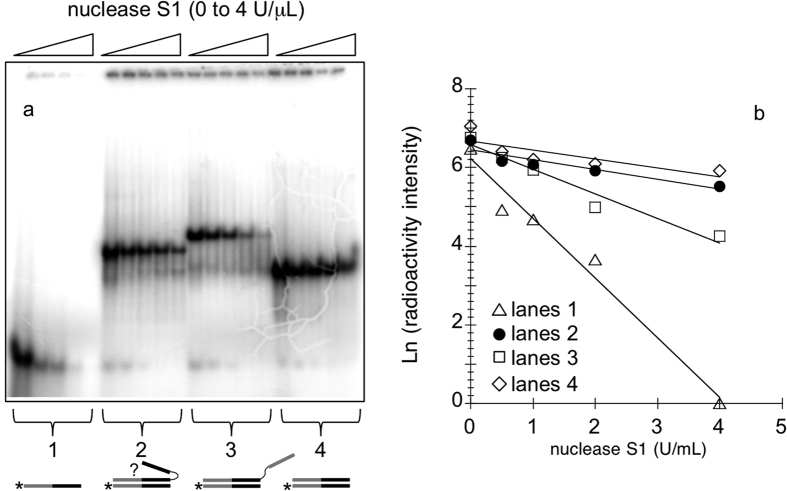
Nuclease S1 assay. (**a**) Non-denaturing PAGE migration pattern of four different DNA constructs after incubation with increasing concentrations of nuclease S1 (from 0 to 4 U/μL). Lanes 1: radiolabelled *Y2rep2*’ (*Y2rep2*’*). Lanes 2: *Y2rep2*’* + *HpE2rep2*. Lanes 3: *Y2rep2*’* + *R2rep2*/*overhang*. Lanes 4: *Y2rep2*’* + *R2rep2*. (**b**) Quantification of the gel shown in (**a**). Nuclease S1 assays were carried out in a buffer containing KCl and MgCl_2_ on DNA samples annealed in the presence of KCl and MgCl_2_.

To further ascertain triplex formation, we carried out a nuclease S1 assay. S1 is an endonuclease that cleaves single-stranded polynucleotide chains. Radiolabelled *Y2rep2*’ (*Y2rep2*’*) was slowly annealed from high to low temperature (in a buffer containing potassium and magnesium) with *HpE2rep2*, or *R2rep2*, or a control oligonucleotide *R2rep2*/*overhang* (Table 1). This control oligonucleotide hybridizes all along *Y2rep2*’ forming a duplex with a single-stranded overhang that cannot form a triplex structure. After annealing, the nuclease S1 was added to each sample at increasing concentrations; the enzymatic reaction was stopped after 7 min and 30 s, and the resulting products were then analysed on a non-denaturing magnesium containing PAGE (Fig. 6a). For each DNA construct, we fitted a linear equation to *Ln(radioactivity intensity*) vs *S1 concentration* (as shown in Fig. 6b). For each couple of DNA constructs, we defined the relative resistance to nuclease S1 degradation as the ratio between the slopes of the corresponding straight lines. We calculated errors (standard deviation) on relative resistances from independent experiments. On one side, the structure formed by *Y2rep2*’ + *HpE2rEp2* was 2.3 + /−0.3 times more resistant to S1 degradation than the control structure formed by *Y2rep2*’ + *R2rep2*/*overhang* (the duplex with a single-stranded overhang); on the other side, *Y2rep2*’ + *HpE2rep2* was roughly as resistant as the control duplex *Y2rep2*’ + *R2rep2* (relative resistance 0.8 +/− 0.1). Both *Y2rep2*’ + *HpE2rep2* and *Y2rep2*’ + *R2rep2* were about 5 times more resistant to S1 degradation than the single-strand *Y6rep6*’. These results show that, in the complex formed by *Y2rep2*’ + *HpE2rep2*, the *HpE2* motif is protected from nuclease S1 degradation, thus supporting the formation of a triplex structure.

In conclusion, altogether UV-melting, EMSA and nuclease assay support that, when *HpE2rep2* and *Y2rep2*’ are annealed together in potassium and magnesium from high to low temperature, they form an extended structure where the *HpE2* is in a triplex state, and that this complex is more stable than the target duplex formed by *Y2rep2*’ and *R2rep2*. Furthermore, EMSA shows that, when a pre-structured *HpE2rep2* is put in the presence of *Y2rep2*’ at a fixed temperature, despite the *HpE2* motif is structured in a stable G4, more than 50% of *HpE2rep2* forms the extended triplex structure (Fig. 5, lane 3).

## Conclusions

**Figure 7:**
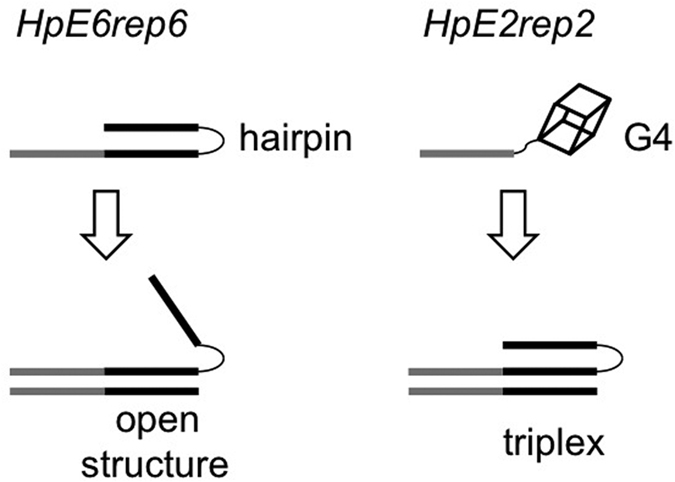
Schematic illustration of the structures formed by *HpE6rep6* and *HpE2rep2* alone and in the presence of their single-stranded targets. The *HpE6* motif alone folds into a reverse Hoogsteen hairpin; it hybridizes to its pyrimidine target sequence; nevertheless, upon hybridization, the hairpin opens. In potassium, the *HpE2* motif folds into a stable G4; in the presence of its target, the *HpE2* motif converts from the G4 conformation into a hairpin conformation, forming a triplex with its pyrimidine target sequence.

PPRH oligonucleotides have been conceived to target polypyrimidine sequences of genomic DNA via the formation of a triplex structure and have been successfully used to knockdown gene expression. Recently, we were able to correct single-point mutations in mammalian cell lines using two PPRH oligonucleotides, each bearing a sequence identical (except for the corrected single-point mutation) to the genomic sequence to be repaired^23^,^24^. In this work, we investigated the structure and the stability of these two repair-PPRH oligonucleotides and of the complexes they form with their single-stranded target sequences. We showed that the *HpE6* motif alone folded into a reverse-Hoogsteen hairpin, while the structure of the G-rich *HpE2* motif depended on the nature of the cation present in solution: in sodium it formed a reverse-Hoogsteen intermolecular duplex; in potassium it folded into a stable intramolecular G4. Nevertheless, the *HpE6* hairpin-prone motif was not able to form a triplex with its single-stranded polypyrimidine target: upon hybridization, the hairpin structure opened, leaving a hanging third strand (Fig. 7). Conversely, the *HpE2* G4-prone motif formed a triplex. In particular, we showed that, upon hybridization of *HpE2rep2* to its target, the *HpE2* motif converted from a stable G4 structure into a reverse-Hoogsteen hairpin and formed a stabilizing triplex with its single-stranded polypyrimidine target (Fig. 7). Our work proves that folding of a PPRH oligonucleotide into a reverse-Hoogsteen hairpin does not necessarily lead to a stable triplex with the target sequence, while folding of a PPRH oligonucleotide into a stable G4 does not necessary impair sequence-specific DNA recognition by triplex formation. Results obtained for these two PPRH oligonucleotides cannot be generalised to other PPRH oligonucleotides. PPRH behaviour is sequence-dependent. For example, while the hairpin-prone *HpE6* does not form a triplex, a different hairpin-prone PPRH oligonucleotide, previously studied, formed a triplex^22^. Since the conformation and stability of G4s are strongly sequence-dependent, we expect the behaviour of G4-prone PPRH oligonucleotides being strongly-sequence dependent as well. Despite their different structural characteristics, both repair-PPRH oligonucleotides *HpE6rep6* and *HpE2rep2* have been proved to be simple and powerful tools to correct point mutations in cells; we are currently investigating which are the structural features that make a PPRH oligonucleotide efficient in targeting genomic DNA.

Our works also illustrates an original example of DNA structural conversion of a G4 into a reverse-Hoogsteen hairpin driven by triplex formation. Such a structural conversion might occur at particular loci of genomic DNA and be involved in genome dynamics or genome instability. Homopurine/homopyrimidine mirror-symmetrical sequences of genomic DNA can potentially fold into intrachromosomal triplex structures (H-DNA)^38^. H-DNA prone sequences are abundant in human genome^39^; many H-DNA prone sequences can potentially fold also in G4s, and are involved in genome instability^40^. An example of transcription arrest caused by triplex formation in a G4/H-DNA prone region has been reported for the nuclease-hypersensitive element NHE III_1_ of the human c-myc promoter^41^. We are currently investigating G4/H-DNA prone sequences in human genome in order to understand if triplex formation may be mediated by G4 formation via a structural conversion similar to the one highlighted in the present work.

## Methods

### Oligonucleotides

Oligonucleotides were purchased from Eurogentec (Belgium). Non-labelled oligonucleotides were Reverse Phase Cartridge•Gold™ purified; the double-dye labelled FAM-*HpE6*-Dabcyl was Reverse Phase HPLC purified. Oligonucleotides were dissolved in bi-distilled water (at a concentration of 200 μM) and stored at −20 °C. Concentrations were determined by ultraviolet light (UV) absorption using molar extinction coefficients provided by the manufacturer. Oligonucleotide sequences are listed in Table 1.

### UV absorption measurements

Oligonucleotides were dissolved in a cacodylic acid buffer (10 mM) at pH 7.0 (adjusted with LiOH), containing NaCl or KCl (100 mM), in the absence or in the presence of MgCl_2_ (10 mM). Oligonucleotide strand concentrations are indicated in figure legends (error on strand concentration was estimated to be about 10%). UV absorbance as a function of temperature was recorded on a XL spectrophotometer (Secomam) according to the following protocol: samples were heated at 95 °C for 2 min, cooled from 95 to 5 °C at a rate of 0.2 °C min^−1^, kept at 5 °C for 10 min, and heated from 5 to 95 °C at a rate of 0.2°Cmin^−1^; the absorbance was recorded at 260, 295 and 335 nm. Temperature was varied with a circulating water bath and measured with an inert glass sensor immersed into a water-filled quartz cell; evaporation at high temperatures and condensation at low temperatures were prevented by a layer of mineral oil and by a dry airflow in the sample compartment, respectively. Melting profiles were corrected for baseline drifting (the absorbance at 335 nm was subtracted from the absorbance at 260 and 295 nm). We defined “temperature of thermal transition”, *T*_t_, the first derivative of the absorbance as a function of the temperature (+/−1 °C uncertainty). Each UV melting experiment was carried out two or three times (at least); melting profiles were perfectly reproducible (perfectly superimposable). Thermal difference spectra (TDS) were obtained by subtracting the absorption spectrum at low temperature (5 °C) from the absorption spectrum at high temperature (95 °C); absorption spectra at low temperature were recorded after annealing the samples from 95 to 5 °C at 0.2 °C min^−1^. Circular dichroism (CD) spectra were recorded on a JASCO-810 spectropolarimeter.

### Fluorescence measurements

Fluorescence measurements were carried on an *HpE6* oligonucleotide bearing a 6-carboxyfluorescein (FAM) at its 5′ extremity and a Dabcyl quencher at its 3′ extremity. For melting experiments, FAM-*HpE6*-Dabcyl (at a final concentration of 0.2 μM) was dissolved in a cacodylic acid buffer (10 mM) at pH 7.0 (LiOH), containing KCl (100 mM) and MgCl_2_ (10 mM), in the absence or in the presence of *Y6* oligonucleotide (2 μM). FAM emission as a function of temperature was recorded on a SPEX Fluorolog (HORIBA Jobin Yvon) at an excitation wavelength of 470 nm and an emission wavelength of 520 nm; temperature was raised with a circulating water bath from 5 to 80 °C at 1 °C min^−1^. FAM-*HpE6*-Dabcyl emission as a function of temperature was normalized between the minimum and the maximum of fluorescence; we defined “temperature of half-dissociation”, *T*_1/2_, the temperature at which the normalized fluorescence was equal to 0.5.

### Polyacrylamide gel electrophoresis (PAGE)

Single-stranded oligonucleotides (*Y6rep6*’, *Y2rep2*’ and *Y2*) were 5′end-labelled with [γ^32^P]ATP using a T4 polynucleotide kinase (NEB). DNA samples were prepared in a cacodylic acid buffer (10 mM) at pH 7.2 (LiOH) containing NaCl or KCl (100 mM) and MgCl_2_ (10 mM) at a strand concentration of about 15 nM of radiolabelled oligonucleotides (*Y6rep6*’, *Y2rep2*’ and *Y2*) and 1.5 μM of non-radiolabelled oligonucleotides (*HpE6rep6, R6rep6, HpE2rep2, R2rep2* and *HpE2*). Sample annealing was carried out as indicated in figure legends. Polyacrylamide gels (12%, acrylamide:bisacrylamide mass ratio of 19:1) were prepared in a TBE buffer, supplemented with NaCl or KCl (20 mM) and with MgCl_2_ (10 mM). Electrophoresis was run in a TBE buffer, supplemented with NaCl or KCl (20 mM) and with MgCl_2_ (10 mM), in a cold room, at 3 W/gel, for about 3 h. The temperature of the gel during migration was about 15 °C. Gels were dried and exposed to Phosphorimager screens and screens were scanned with a Typhoon 9410 Imager (Molecular Dynamics). In PAGE experiments shown in Fig. 2c and in Fig. S5, oligonucleotides were not radiolabelled and they were detected by UV-shadow at 254 nm with a G:BOX (Syngene).

### Nuclease S1 assays

Radiolabelled *Y2rep2*’ (*Y2rep2*’*) were slowly annealed from high to low temperature (4 °C) in the presence of *HpE2rep2*, or *R2rep2*, or *ctr-dx*/*ss* oligonucleotides, in a cacodylic acid buffer (10 mM) at pH 7.2 (LiOH) with KCl (100 mM) and MgCl_2_ (10 mM), at a strand concentration of 110 nM of *Y2rep2*’* and 500 nM of *HpE2rep2*, or *R2rep2*, or *ctr-dx*/*ss*. Strand hybridization was checked on a polyacrylamide gel (4%, acrylamide:bisacrylamide mass ratio of 19:1) made in TBMg 0.5× buffer (44.5 mM Tris-Base, 44.5 mM boric acid, 5 mM MgCl_2_). The DNA samples were next diluted in the Nuclease S1 buffer (25 mM Tris HCl pH 7.5, 50 mM KCl, 20 mM MgCl_2_ and 5% glycerol) to reach a final concentration of 0.5–1 nM. Samples (20 μL) were prewarmed for 5 minutes at 25 °C. The Nuclease S1 was added at the concentrations indicated in the figure legend. After 7 min and 30 s at 25 °C, the samples were put on ice to stop the reaction, supplemented with glycerol (4%) and immediately loaded on a polyacrylamide gel (12%, acrylamide:bisacrylamide mass ratio of 19:1) made in TBMg 0.5×. Electrophoresis was performed at 4 °C, in TBMg 0.5× and at 150 V for 4 hours. After electrophoresis, the gel was dried and exposed on a Phosphorimager screen. After being exposed for at least 10 h, the screen was scanned with a Typhoon 9410 Imager (Molecular Dynamics). ImageQuant (version 5.1) was used to quantify the gels. Nuclease assays were repeated at least twice.

## Additional Information

**How to cite this article:** Solé, A. *et al*. Polypurine reverse-Hoogsteen (PPRH) oligonucleotides can form triplexes with their target sequences even under conditions where they fold into G-quadruplexes. *Sci. Rep.* **7**, 39898; doi: 10.1038/srep39898 (2017).

**Publisher's note:** Springer Nature remains neutral with regard to jurisdictional claims in published maps and institutional affiliations.

## Supplementary Material

Supplementary Information

## Figures and Tables

**Figure 1:**
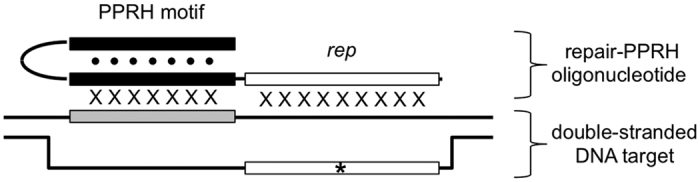
Schematic illustration of a PPRH-repair oligonucleotide and its genomic DNA target. Repair-PPRH oligonucleotides are composed of a PPRH motif (black segments) and of a single-stranded *rep* extension (white segment) homologous to the genomic sequence to be replaced (white segment on DNA target; the asterisk symbol represents a single-point mutation to be corrected). A PPRH motif is designed to fold, in principle, into a reverse-Hoogsteen hairpin and to form a triplex with its genomic polypyrimidine target sequence (grey segment); points and crosses represent reverse-Hoogsteen and Watson-Crick base-pairs, respectively. When the double-stranded DNA target is in an open-state, as represented in this figure, the *rep* sequence can hybridize, via Watson-Crick base-pairs, to the single-stranded region next to the PPRH target.

**Table 1:**
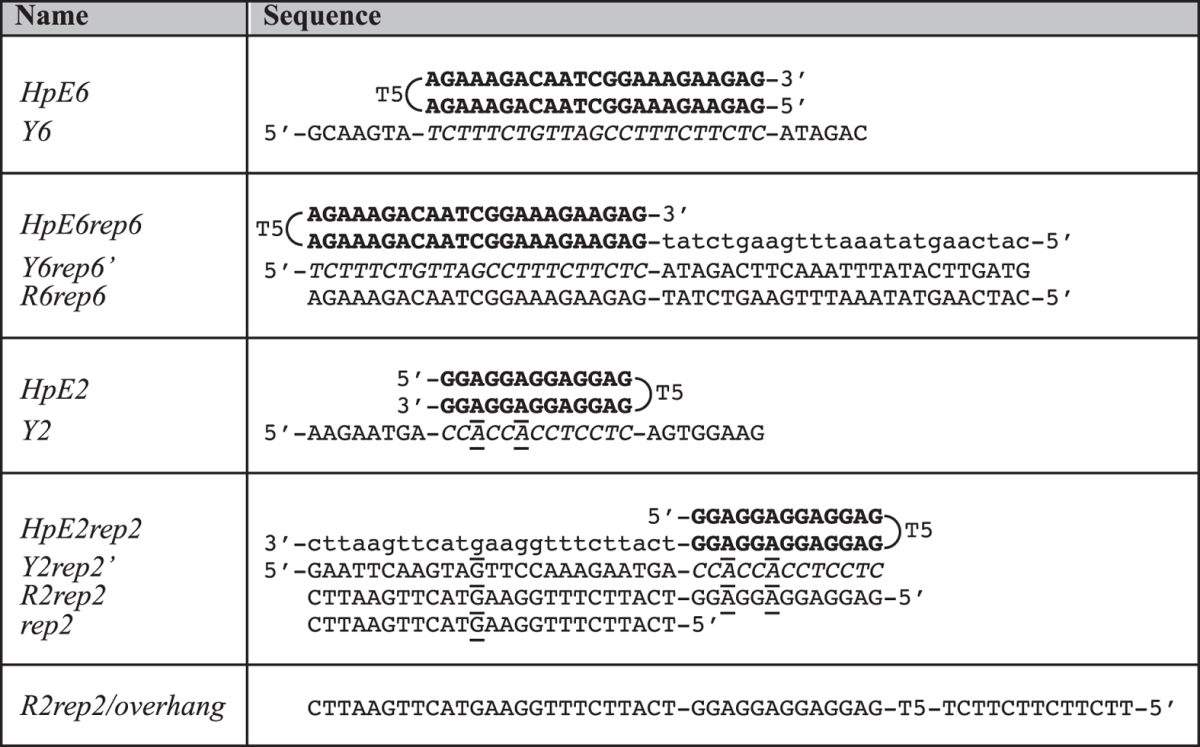
Oligonucleotides sequences used in this study.

*HpE6rep6* and *HpE2rep2* are composed of a PPRH motif (*HpE6* and *HpE2* domain, bold font) and of a 25 nucleotide single-stranded extension (*rep6* and *rep2* domain, lower case letters).*Y6rep6*’ and *Y2rep2*’ are the single-stranded targets of *HpE6rep6* and *HpE2rep2*, while *R6rep6* and *R2rep2* are the complementary sequences of *Y6rep6*’ and *Y2rep2*’.In this table, *HpE6* and *HpE2* motifs are represented in a reverse-Hoogsteen hairpin conformation; their pyrimidine target sequences are in italic font.Underlined letters represents Watson-Crick base-pair mismatches. *R2rep2/overhang* is a control oligonucleotide used in nuclease S1 assays.
