# Supplementary material for: Polypurine reverse-Hoogsteen (PPRH) oligonucleotides can form triplexes with their target sequences even under conditions where they fold into G-quadruplexes
Source: Sci Rep. 2017 Jan 9;7:39898. doi: 10.1038/srep39898 (PMC5220335; doi:10.1038/srep39898)
Supplement: Supplementary Information [file srep39898-s1.pdf]

Supplementary Information

Polypurine reverse-Hoogsteen (PPRH) oligonucleotides can form triplexes with their target sequences even under conditions where they fold into G-quadruplexes.

*Anna Solé [a], Emmanuelle Delagoutte [b], Carlos J. Ciudad [a], Véronique Noé [a], Patrizia Alberti [b]**

[a] Departement of Biochemistry and Physiology , School of Pharmacy,
University of Barcelona, Barcelona, Spain

[b] Structure et Instability of Genomes,
Sorbonne Universités, Muséum national d'Histoire naturelle,
Inserm U 1154, CNRS UMR 7196, Paris, France

* alberti@mnhn.fr

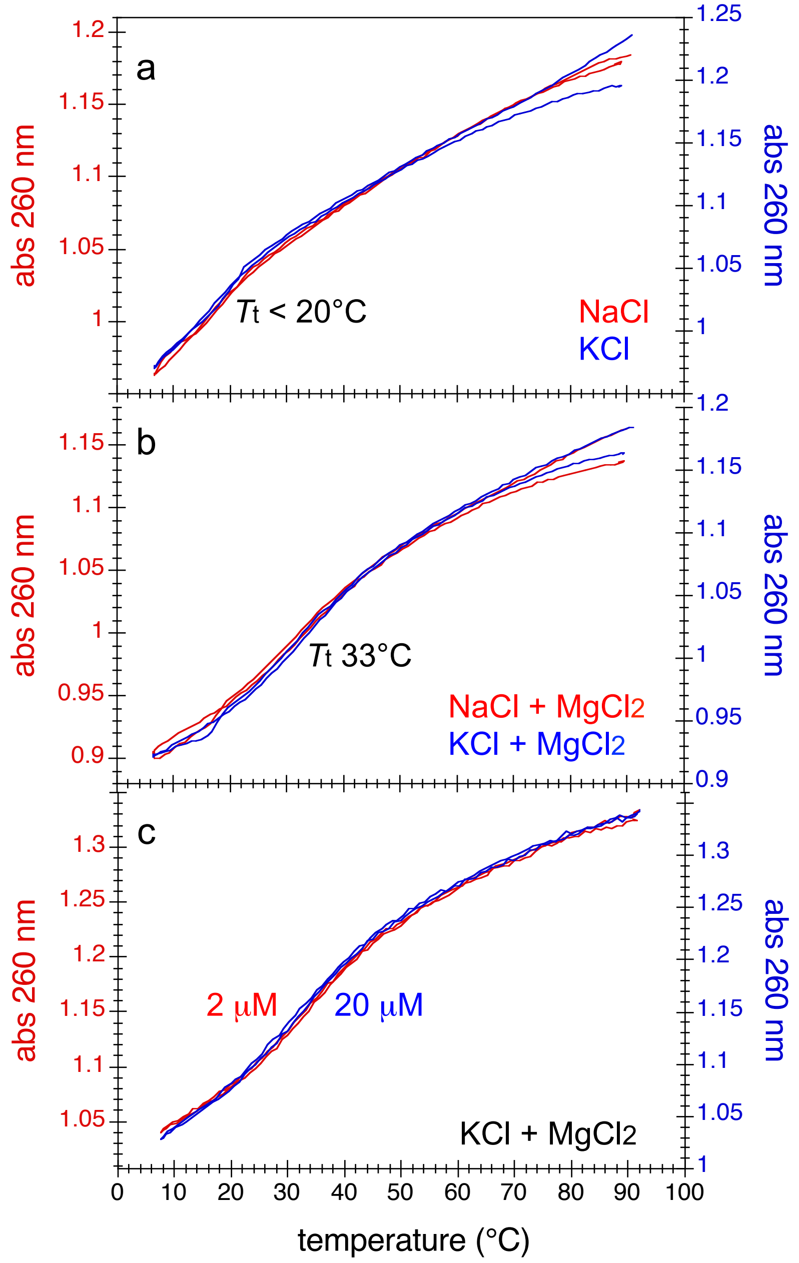

Figure S1
UV-melting profiles of *HpE6* oligonucleotide

Absorbance at 260 nm as a function of temperature of
(a) 2µM *HpE6* in 100 mM NaCl (red) and in 100 mM KCl (blue),
(b) 2 µM *HpE6* in 100 mM NaCl + 10 mM $MgCl_2$ (red) and in 100 mM KCl + 10 mM $MgCl_2$ (blue).
(c) 2 µM (red) and 20 µM (blue) *HpE6* in 100 mM KCl + 10 mM $MgCl_2$.
The optical path length was 10 mm for 2µM *HpE6* and 1 mm for 20 µM *HpE6*.
Both cooling and heating profiles are shown.

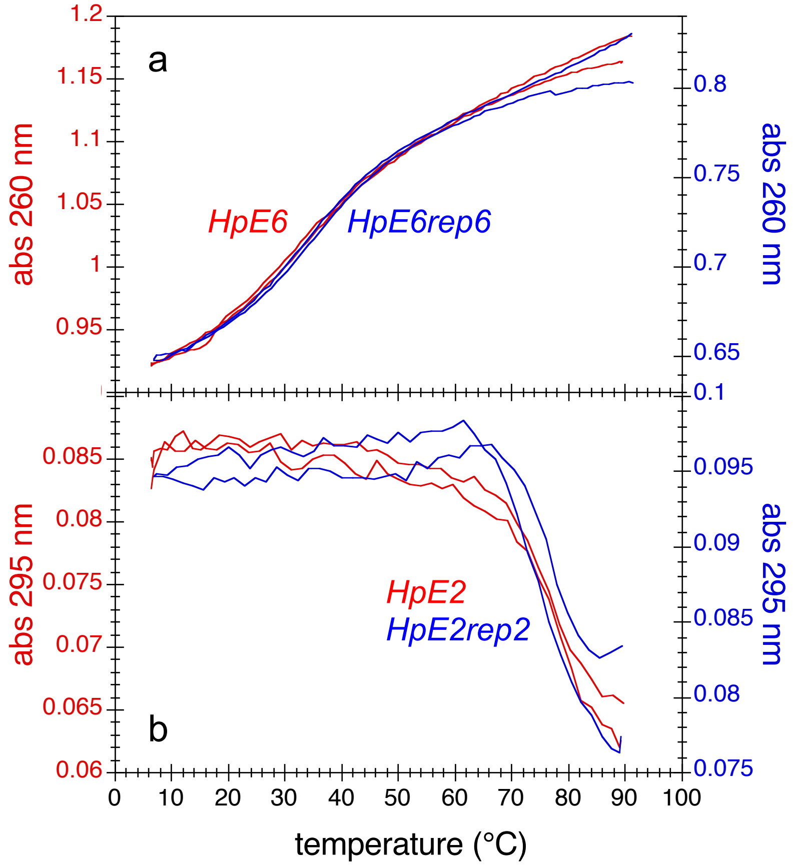

Figure S2
UV-melting profiles of *HpE6(rep6)* and *HpE2(rep2)* oligonucleotides

(a) Absorbance at 260 nm as a function of temperature of *HpE6* (red) and *HpE6rep6* (blue).
(b) Absorbance at 295 nm as a function of temperature of *HpE2* (red) and *HpE2rep2* (blue).
Measurements were carried out at 2 μM strand concentration for *HpE6* and *HpE2* and at 1.5 μM strand concentration for *HpE6rep6* and *HpE2rep2*, in a buffer containing 100 mM KCl and 10 mM $MgCl_2$.
Both cooling and heating profiles are shown.

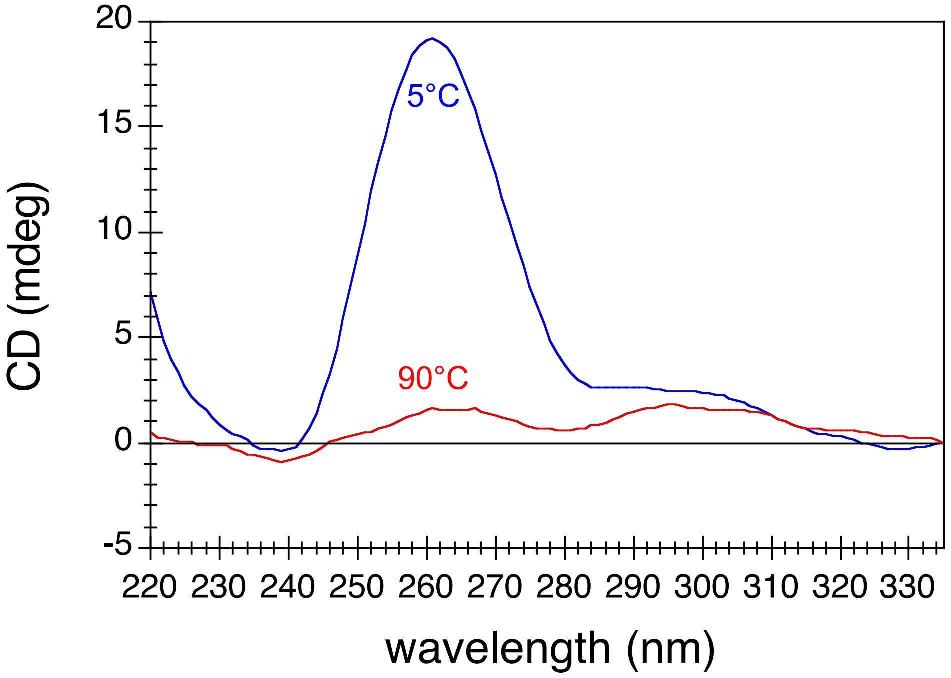

Figure S3

CD spectra of *HpE2* oligonucleotide in potassium and magnesium

Circular dichroism spectra of 2 μM *HpE2* in 100 mM KCl + 10 mM $MgCl_2$, at 5°C (blue) and 90°C (red).

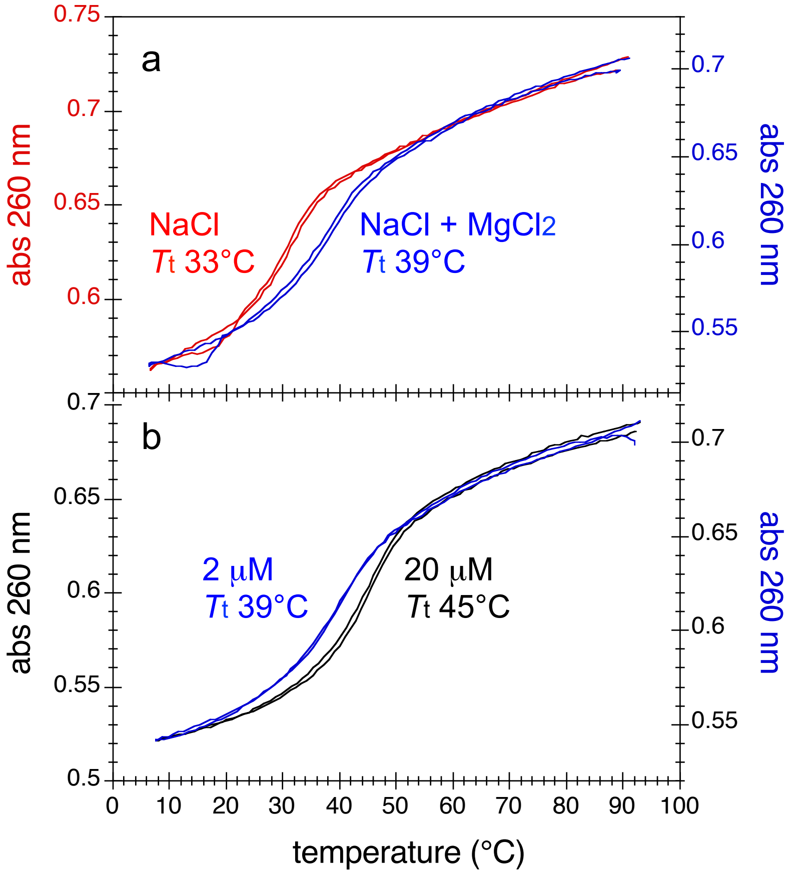

Figure S4
UV-melting profiles of *HpE2* oligonucleotide in sodium

(a) Absorbance at 260 nm as a function of temperature of 2 µM *HpE2* in 100 mM NaCl (red) and in 100 mM NaCl + 10 mM $MgCl_2$ (blue).

(b) Absorbance at 260 nm as a function of temperature of *HpE2* in 100 mM NaCl + 10 mM $MgCl_2$, at 2 µM (blue) and 20 µM (black) strand concentration.

Both cooling and heating profiles are shown.

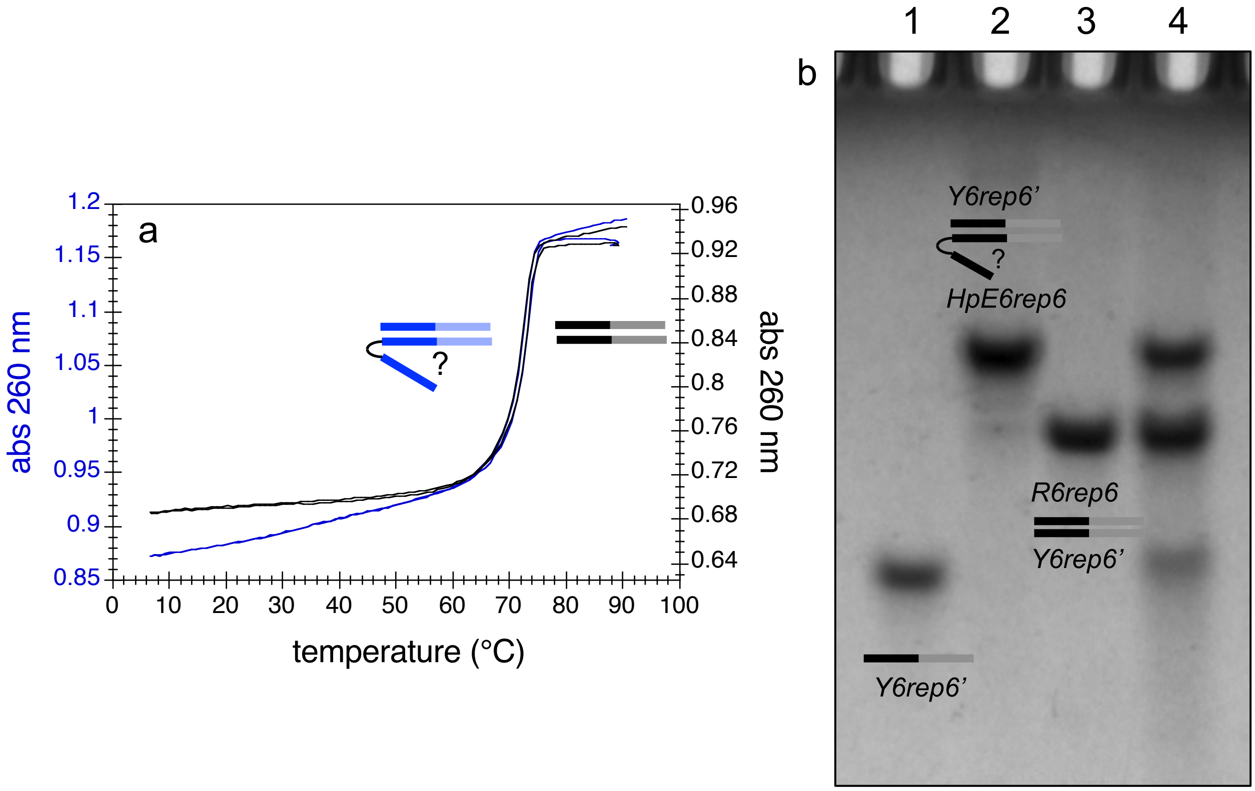

Figure S5
UV-melting profiles and PAGE of the complex *HpE6rep6 + Y6rep6’*

(a) Absorbance at 260 nm as a function of temperature of 1 µM *Y6rep6’* + 1 µM *HpE6rep6* (red) and of 1 µM *Y6rep6’* + 1 µM *R6rep6* (black), in 100 mM KCl + 10 mM $MgCl_2$.
Both cooling and heating profiles are shown.

(b) Non-denaturing PAGE of :
Lane 1: *Y6rep6’ 25 µM*;
lane 2: *HpE6rep6 25 µM* + *Y6rep6’ 25 µM*;
lane 3: *Y6rep6’ 25 µM* plus its complementary strand *R6rep6 25 µM*;
lane 4: *HpE6rep6 25 µM* + *Y6rep6’ 25 µM* + *R6rep6 25 µM*.
Samples were prepared in a buffer containing 100 mM KCl + 10 mM $MgCl_2$, heated at 90°C for 2 min and slowly cooled at 4°C. The gel and the migration buffer contained 20 mM KCl + 10 mM $MgCl_2$. Oligonucleotides were detected by UV-shadow. Identical UV melting profiles and PAGE patterns were obtained in 100 mM NaCl + 10 mM $MgCl_2$.

The UV-melting profile of *Y6rep6’* + *HpE6rep6* is similar to the one of the duplex formed by *Y6rep6’* and its complementary strand *R6rep6*; both exhibit a single melting transition at 73°C. This demonstrates that the *HpE6rep6* forms a duplex with *Y6rep6’*, but does not allow inferring triplex formation by the *HpE6* motif.
In non-denaturing PAGE, annealing of *Y6rep6’* in the presence of both *HpE6rep6* and *Rrep6* results in the formation of two bands of similar intensities (lane 4), one corresponding to the duplex formed by *Y6rep6’* + *R6rep6* (in lane 3), the other migrating as the complex formed by *Y6rep6’* + *HpE6rep6* (in lane 2). The fact that the two bands have similar intensities indicates that the structure formed by *HpE6rep6* with its target *Y6rep6’* is as stable as the duplex formed by *Y6rep6’* with its complementary strand *R6rep6*.

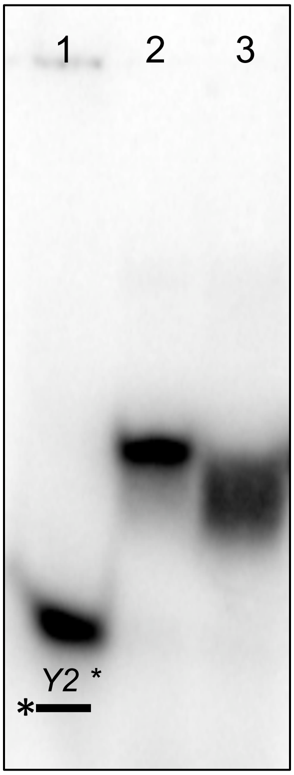

Figure S6
EMSA of the complex *HpE2* + *Y2* in potassium and magnesium

Electrophoretic mobility shift assay of the *HpE2* system in KCl + $MgCl_2$.
Lane 1: radiolabeled *Y2* strand (*Y2**);
lane 2: *Y2** + *HpE2*; the mix was heated at 95°C and slowly annealed at 5°C;
lane 3: *HpE2* was heated at 95°C and slowly annealed at 5°C, then *Y2** was added.
EMSA was carried out as described in the Material and Methods section.

Annealing of *HpE2* with *Y2** from high to low temperature resulted in a single thin band (lane 2), supporting the formation of a single complex (a Watson-Crick duplex with a hanging third strand or a triplex?); while incubation of *Y2** with a structured *HpE2* (separately annealed alone in the presence of potassium) resulted in a smear band supporting the presence of multiple conformational states.

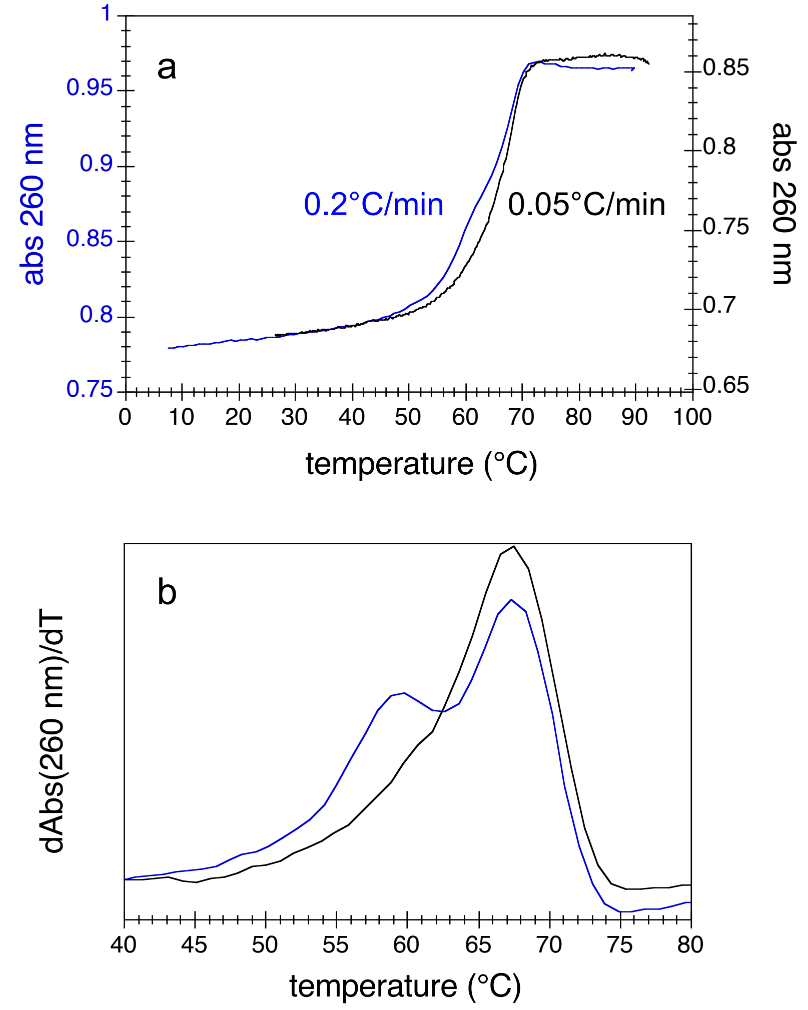

Figure S7
UV-annealing profiles of the complex(es) formed by *HpE2rep2* and *Y2rep2’* in potassium and magnesium at different temperature scanning rates

(a) Absorbance at 260 nm as a function of temperature from 95°C to 5°C of *Y2rep2’* + *HpE2rep2* at a scanning rate of 0.2°C/min (blue) and of 0.05°C/min (black).

(b) First derivative of absorbance as a function of temperature of the annealing profiles shown in (a).

Each oligonucleotide were at 1µM strand concentration. Measurements were run in a buffer containing 100 mM KCl and 10 mM $MgCl_2$.
